# Thermoelectric Energy Equations Considering Convective Heat Transfer Between Thermoelectric Materials and the Environment

**DOI:** 10.3390/ma18040859

**Published:** 2025-02-16

**Authors:** Heng Xiao

**Affiliations:** 1 Key Laboratory of Fluid and Power Machinery, Ministry of Education, Xihua University, Chengdu 610039, China; xh-840822@163.com or xiaoheng@xhu.edu.cn; 2 Key Laboratory of Fluid Machinery and Engineering, Sichuan Province, Xihua University, Chengdu 610039, China

**Keywords:** thermoelectric generator, thermoelectric energy equation, convective heat dissipation, linear temperature distribution

## Abstract

Thermoelectric power generation is increasingly becoming a research field with practical application value and broad application prospects. Many researchers and engineers have used the classic thermoelectric energy equations in the analysis of thermoelectric systems. However, it is assumed that the thermoelectric material is thermally insulated from its surroundings except at the junctions of the hot and cold ends where heat enters and leaves. Based on a detailed study of the classic thermoelectric effects and heat transfer theory, the revised thermoelectric energy equations are derived, which take into account the convective heat dissipation from the surface of thermoelectric materials to the ambient. The revised equations have a very simple form, which is very convenient for engineering calculation and analysis. A comparison of the results of formula calculation and numerical simulation was conducted to verify the reasonableness of the linear temperature distribution assumption in the derivation process. Within the computational range of this study, the differences between the simulated values and the calculated values are controlled to be a little more than 1%, which is sufficient to meet the needs of engineering calculation and analysis.

## 1. Introduction

Research on thermoelectric materials has been in full swing in recent years, and much progress has been made at the laboratory level. However, thermoelectric materials are ultimately intended for applications, particularly thermoelectric cooling and thermoelectric power generation. In response to the serious and imminent crisis posed by global climate change, countries around the world are seeking to move away from fossil fuels and adopt new energy technologies on a large scale. China, in particular, is pushing for ambitious targets of so-called carbon peaking and carbon neutrality. Thermoelectric (TE) power generation, as an all-solid-state energy conversion technology with no fluid working medium and no transport piping, naturally has special and even significant advantages in certain specific applications. The thermoelectric generator’s (TEG) compact design and lack of rotating parts make it quiet, reliable, and virtually maintenance free. The absence of chemical fluids and lubricants to protect the rotating parts makes the thermoelectric generator very environmentally friendly [1]. Based on the above technical advantages and policy background, and taking into account the traditional demand for energy conservation and emission reduction, the thermoelectric power generation technology, which uses thermoelectric materials to directly convert thermal energy into electrical energy, is increasingly becoming a research field with practical application value and broad application prospects [2].

However, the promotion and application of TEG is limited by the low thermoelectric conversion efficiency of current TE materials [3]. Bell [4] pointed out that there are two ways to increase the adoption of thermoelectric devices. One is to make a breakthrough in materials research, i.e., to improve the efficiency of thermoelectric materials at the application level, not just at the laboratory level. The other is to start at the system level, i.e., how to use, integrate, and optimize thermoelectric components. Research on thermoelectric materials has increased in recent years, but it has been difficult to make major breakthroughs at the application level for the near future. It is more pragmatic for researchers and engineers in the energy sciences to focus on how thermoelectric materials and technologies can be used to improve the efficiency of the entire thermoelectric conversion device at the system level. As a typical case, Chen [5] proposed the idea of integrating TEGs with combined heat and power (CHP) units. In this way, the thermal energy is used twice in the original CHP system, or more precisely, the gradient utilization of thermal energy is realized, thus improving the overall efficiency of energy utilization.

In addition to the development of the most fundamental and core thermoelectric materials and the application of thermoelectric components as key to their technical realization, there has been considerable research in recent years into the analysis and optimization of the performance of thermoelectric systems, involving knowledge of materials science, thermodynamics, heat transfer, and fundamental physics. As representative studies of a slightly earlier period, Rowe [6] developed a computational program to evaluate the performance of a commercial thermoelectric module as a core component of a TEG based on classic thermoelectric theory and related thermophysical theories, while Chen [7] used the theory of irreversible process thermodynamics to study the performance of TEGs with internal and external irreversibilities. In both of these classic studies, the researchers used thermoelectric generators as devices consisting of a single TE material (thermocouple or thermoelectric pair). However, actual thermoelectric modules (especially commercial ones) are composed of several TE pairs. In order to reconcile theory with practice, thermoelectric generators should be studied as multi-component devices rather than as one-piece devices. Therefore, Chen [8,9] and Pan [10] conducted systematic analysis and optimization studies of multi-component thermoelectric generators.

For a long time, many researchers and engineers have used what the author calls the classic thermoelectric energy equations in the analysis and optimization of thermoelectric devices and systems [11,12] and in engineering calculations [13,14]. Even in the performance optimization and analysis of a thermoelectric refrigeration system [15] and a photovoltaic thermoelectric cogeneration system [16], and in the modeling of a thermoelectric generator applied to a vehicle [17] or an internal combustion engine [18] waste heat recovery, the classic thermoelectric energy equations remain the basis of the work performed. The classic thermoelectric energy equations take into account the irreversibility within the thermoelectric component due to Joule heating and heat conduction within the thermoelectric material. However, it is assumed that the thermoelectric material is thermally insulated from its surroundings, i.e., there is no heat exchange between the surface of the thermoelectric material and the ambient, except at the junctions of the hot and cold ends where heat enters and leaves [12]. In two recent reviews of advances in thermoelectric power generation, it can be seen that the classic thermoelectric energy equations remain one of the most important theoretical foundations for the modeling and simulation of thermoelectric generators [19]. Although a number of researchers have proposed some improved models, they are all based on the basic form of the classic thermoelectric energy equations, even if the Thomson effect, which is completely negligible in practice, is taken into account [20].

Since today’s commercial thermoelectric modules consist of multiple TE pairs, and the TEGs are usually integrated by multiple thermoelectric modules, whether to consider the heat exchange between the ambient and the TE pairs is a realistic issue to be evaluated. Most commercial thermoelectric modules are sufficiently small to be thermally insulated around the TE materials. However, some customized TEGs are encapsulated with large-sized TE materials. In such cases, the heat exchange between the TE material and its surroundings must be taken into account to ensure the accuracy of the calculations, especially for medium- to high-temperature applications. Given the numerous factors influencing the convective heat transfer coefficient, it is imperative to recognize that thermoelectric materials of varying configurations and sizes are also subject to varying degrees of convective heat transfer. This underscores the necessity for systematic and targeted research in this field. In this paper, based on a detailed study of the classic thermoelectric effects and heat transfer theory, what the author calls the revised thermoelectric energy equations are derived, which take into account the convective heat transfer between the ambient and the TE materials.

## 2. System Modeling and Equations Derivation

**Figure 1:**
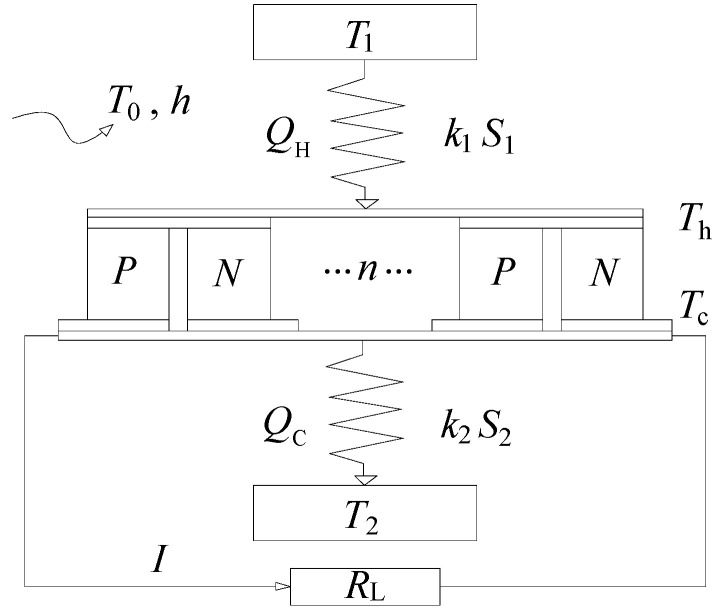
Schematic representation of a multi-component thermoelectric generator.

As shown in Figure 1, a typical TEG consists of multiple TE pairs operating between a high temperature reservoir (*T*_1_) and a low temperature reservoir (*T*_2_). Each TE pair consists of a P-type thermoelectric material and an N-type thermoelectric material. All of the TE pairs are connected in parallel or/and series by a circuit and encapsulated into a thermoelectric module by a special construction and process, with a load resistance, *R*_L_, connected in series.

The Peltier effect is a fundamental thermoelectric effect, often considered to be the basis of thermoelectric refrigeration, but it is also the basis of thermoelectric power generation, although the effect is more indirect. The Peltier effect is generally believed to occur at the junctions of thermoelectric pairs, i.e., at the junctions where heat absorption or discharge occurs. It is characterized by the rate of heat absorption at the hot junction (*Q*_ph_) and the rate of heat discharge at the cold junction (*Q*_pc_). It is imperative to acknowledge that, although the Peltier effect is believed to occur at the junctions of thermoelectric pairs, the heat flow resulting from the Peltier effect is observed throughout the thermoelectric leg itself. In technical analyses, the Peltier effect is often expressed as a separate phenomenon occurring at the junctions. The expressions for the Peltier effect are as follows,

(1) $$Q_{\mathrm{ph}}=\alpha IT_{h},$$

(2) $$Q_{\mathrm{pc}}=\alpha IT_{c},$$

where $\alpha_{P}$ and $\alpha_{N}$ are parameters characterizing the thermoelectric conversion capability of P-type and N-type TE materials, respectively, i.e., the Seebeck coefficients, and $\alpha =\alpha_{P}-\alpha_{N}$. *I* is the electric current, an important driving force in the energy transport process, and the driving force leading to the Peltier effect, and *T*_h_ and *T*_c_ denote the temperatures at the hot and cold sides of the thermoelectric generator.

**Figure 2:**
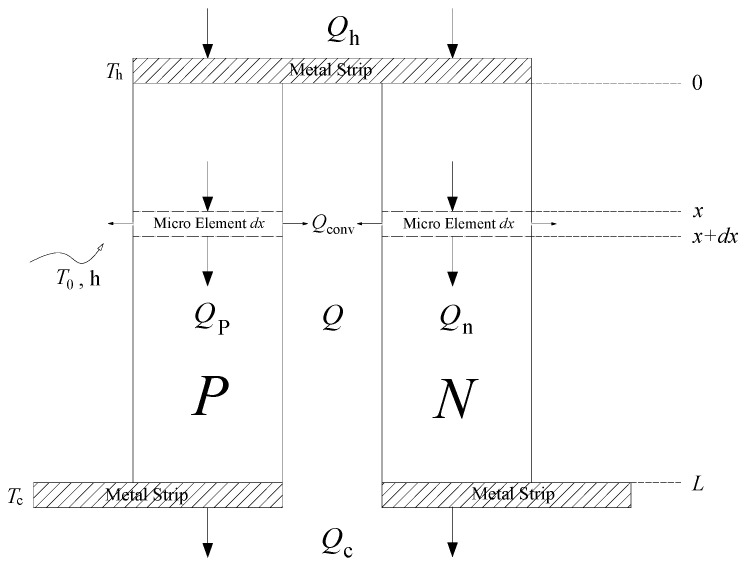
Schematic diagram of a single thermoelectric pair.

Next, a single thermoelectric pair is introduced and analyzed, as shown in Figure 2. The heat flow rates (excluding the heat flow rate resulting from the Peltier effect) in P-type and N-type TE materials are denoted by *Q*_p_ and *Q*_n_, respectively. *k*_p_ (*k*_n_) and $\rho_{p}$($\rho_{n}$) denote the thermal conductivities and the electrical resistivities. The physical parameters of TE materials can usually be assumed to be independent of temperature, as long as the operating temperature range is not too wide. The production of commercial thermoelectric modules is industrialized and scaled up, so that thermoelectric materials are prepared and cut to standard sizes. Consequently, the length (denoted by *L*) and other dimensions of each TE leg (two legs make a pair) are assumed to be the same. In fact, P-type TE materials and N-type TE materials are dimensionally identical, and the cross-sectional area of the TE leg is denoted by *S*_p_ (*S*_n_).

The heat loss from the surface of the thermoelectric materials to the ambient will be considered in the following work. A preliminary discussion of the heat transfer between the thermoelectric legs and the ambient can be found in the authors’ previous work [21]. A recent theoretical study has provided a detailed derivation of the thermoelectric energy equations, but the results obtained are not entirely accurate [22], where the assumption of a linear temperature distribution was also employed. However, the mathematical techniques utilized were rudimentary, akin to a ‘patchwork’ of physical concepts, resulting in a concise yet not rigorous conclusion. Reference can also be made to relevant theoretical analyses [23] and experimental studies [24].

Therefore, in accordance with the principle of conservation of energy, the steady state energy control equation for an infinitesimal cell (a micro-element of length *d*x) of a single TE leg (P-type) can be written as follows,

(3) $$Q_{p}(x)+I^{2}\frac{\rho_{p}}{S_{p}}dx=Q_{p}(x+dx)+hP[T_{p}(x)-T_{0}]dx.$$

Equation (3) can be changed to

(4) $$dQ_{p}(x)=Q_{p}(x+dx)-Q_{p}(x)=I^{2}\frac{\rho_{p}}{S_{p}}dx-hP[T_{p}(x)-T_{0}]dx,$$

where *h* represents the coefficient of convective heat transfer, *T*_0_ is a constant value of the ambient temperature, and *P* represents the circumference of a single thermoelectric leg. *T*_p_ and *Q*_p_ denote the temperature and the heat flow rate (by conduction) inside the TE leg, which vary with the coordinate *x*. The leftmost term in Equation (4) is the net increase in heat flow rate from position *x* to *x* + *dx*. The two rightmost terms refer to the rates of Joule heating and convective heat loss, which are equivalent to acting as a heat source and a heat sink, respectively.

It is important to note that the convective heat transfer occurs on the side of the thermoelectric leg (lateral heat dissipation), resulting in the entire thermoelectric energy conversion process that seems like it should be treated as a two-dimensional problem. The convective heat transfer term above is, in fact, regarded as an internal heat source term (the term is negative because it signifies heat dissipation), whereas the Joule heat term is regarded as a positive internal heat source term. The aforementioned treatment facilitates the resolution of complex two-dimensional problems in a manner analogous to the resolution of one-dimensional problems. This approach constitutes a classical treatment in the field of heat transfer, particularly with regard to the analysis of heat transfer from extended surfaces [25].

Integrating Equation (4) from 0 to *x* yields

(5) $$Q_{p}(x)-Q_{p}(0)=I^{2}\frac{\rho_{p}}{S_{p}}x-hP\int_{0}^{x}[T_{p}(x)-T_{0}]dx.$$

Although in operation, the temperature inside the thermoelectric leg cannot be linearly distributed due to the presence of thermoelectric conversion and Joule heating. Considering the ease of integration processing (especially in this work it is hoped to derive a more concise formula that is convenient for engineering calculations) and the fact that the error should be within acceptable limits when the temperature difference is not large, a linear distribution of *T*_p_*(x)* is assumed here. In subsequent studies, a comparison of the results of mathematical calculations (based on the assumption of linear temperature distribution) and numerical simulations is necessary to verify the reasonableness of this assumption. It is imperative to assess the discrepancy between the two results for various thermoelectric materials and/or distinct operating temperatures, so as to quantitatively ascertain the extent to which the aforementioned “within acceptable limits” are met. As a result, a specific expression for *T*_p_*(x)* assuming a linear distribution can be obtained:

(6) $$T_{p}(x)=ax+b\;\;(0\leq x\leq L),$$

where the coefficients a and b can be obtained by substituting the following boundary conditions:

(7) $$x=0:\;\;T_{p}(0)=T_{h},$$

(8) $$x=L:\;\;T_{p}(L)=T_{c}.$$

It is easy to derive that the indefinite integral of the temperature distribution *T*_p_(*x*) can be expressed as

(9) $$\int_{0}^{x}T_{p}(x)dx=\int_{0}^{x}(\frac{T_{c}-T_{h}}{L}x+T_{h})dx\;\;(0\leq x\leq L).$$

Then, the following equations can be obtained by bringing the boundary condition for *x* = *L* into Equation (5),

(10) $$Q_{p}(x)-Q_{p}(0)=I^{2}\frac{\rho_{p}}{S_{p}}x-hP\int_{0}^{x}(\frac{T_{c}-T_{h}}{L}x+T_{h}-T_{0})dx==I^{2}\frac{\rho_{p}}{S_{p}}x-hP(\frac{T_{c}-T_{h}}{2L}x^{2}+T_{h}x-T_{0}x),$$

(11) $$Q_{p}(L)=Q_{p}(0)+I^{2}\frac{\rho_{p}}{S_{p}}L-hP(\frac{T_{c}-T_{h}}{2L}L^{2}+T_{h}L-T_{0}L)=Q_{p}(0)+I^{2}\frac{\rho_{p}}{S_{p}}L-hPL(\frac{T_{c}+T_{h}}{2}-T_{0}).$$

It is easy to see that the second term on the right-hand side of the equals sign in Equation (11) is equal to the total amount of heat generated by the Joule effect in a P-type thermoelectric leg and the third term is equal to the total amount of heat dissipated by convection. Since the temperature inside the TE leg is assumed to be linearly distributed, the third term can be equated to the total convective heat transfer between the surface of the TE leg, where the temperature is equal to (*T*_h_ + *T*_c_)/2 at all locations, and the ambient temperature of *T*_0_.

Fourier’s law is an important expression that describes the quantitative relationship between the heat flow rate and the temperature gradient in heat conduction:

(12) $$Q_{p}(x)=-k_{p}S_{p}\frac{dT_{p}(x)}{dx}.$$

Thus, substituting the expression of Fourier’s law into Equation (10) yields

(13) $$k_{p}S_{p}\frac{dT_{p}(x)}{dx}+Q_{p}(0)+I^{2}\frac{\rho_{p}}{S_{p}}x-hP(\frac{T_{c}-T_{h}}{2L}x^{2}+T_{h}x-T_{0}x)=0.$$

Integrating Equation (13) from 0 to *x* yields

(14) $$\int_{0}^{x}k_{p}S_{p}dT_{p}(x)+\int_{0}^{x}Q_{p}(0)dx+\int_{0}^{x}I^{2}\frac{\rho_{p}}{S_{p}}xdx-hP\int_{0}^{x}(\frac{T_{c}-T_{h}}{2L}x^{2}+T_{h}x-T_{0}x)dx=0,$$

(15) $$k_{p}S_{p}[T_{p}(x)-T_{p}(0)]+Q_{p}(0)x+\frac{I^{2}}{2}\frac{\rho_{p}}{S_{p}}x^{2}-hP(\frac{T_{c}-T_{h}}{6L}x^{3}+\frac{T_{h}}{2}x^{2}-\frac{T_{0}}{2}x^{2})=0.$$

Consider the boundary conditions of the hot and cold sides of the TEG,

(16) $$k_{p}S_{p}(T_{c}-T_{h})+Q_{p}(0)L+\frac{I^{2}}{2}\frac{\rho_{p}}{S_{p}}L^{2}-hP(\frac{T_{c}-T_{h}}{6L}L^{3}+\frac{T_{h}}{2}L^{2}-\frac{T_{0}}{2}L^{2})=0.$$

After simple variations, the following equation is obtained,

(17) $$Q_{p}(0)=\frac{k_{p}S_{p}}{L}(T_{h}-T_{c})-\frac{I^{2}}{2}\frac{\rho_{p}}{S_{p}}L+\frac{hPL}{2}(\frac{T_{c}+2T_{h}}{3}-T_{0}).$$

For a more concise presentation, Equation (17) is rewritten as

(18) $$Q_{p}(0)=K_{p}(T_{h}-T_{c})-0.5I^{2}R_{p}+0.5hPL(\frac{T_{c}+2T_{h}}{3}-T_{0}),$$

where *K*_p_ and *R*_p_ denote, respectively, thermal conductance and electric resistance of P-type TE leg, and

(19) $$K_{p}=\frac{k_{p}S_{p}}{L},$$

(20) $$R_{p}=\frac{\rho_{p}}{S_{p}}L.$$

Consequently, the heat flow rate at position *L* can be derived from Equation (11) combined with Equation (18),

(21) $$Q_{p}(L)=K_{p}(T_{h}-T_{c})+0.5I^{2}R_{p}-0.5hPL(\frac{2T_{c}+T_{h}}{3}-T_{0}).$$

The expression for the Fourier part of heat flow rate *Q*_n_ in an N-type thermoelectric leg (excluding the heat flow rate resulting from the Peltier effect) is formally identical to that in a P-type TE leg. Therefore, the equation for a thermoelectric generator consisting of a single thermoelectric pair is

(22a) $$Q(0)=Q_{p}(0)+Q_{n}(0)=K(T_{h}-T_{c})-0.5I^{2}R+hPL(\frac{T_{c}+2T_{h}}{3}-T_{0}),$$

(22b) $$Q(L)=Q_{p}(L)+Q_{n}(L)=K(T_{h}-T_{c})+0.5I^{2}R-hPL(\frac{2T_{c}+T_{h}}{3}-T_{0}),$$

where

(23) $$K=\frac{k_{p}S_{p}}{L}+\frac{k_{n}S_{n}}{L},$$

and

(24) $$R=\frac{\rho_{p}}{S_{p}}L+\frac{\rho_{n}}{S_{n}}L.$$

After taking into account the Peltier effect, expressed by Equations (1) and (2), at both ends of the TEG, the revised thermoelectric energy equations for a thermoelectric generator consisting of a single TE pair can be obtained,

(25a) $$Q_{h}=Q_{\mathrm{ph}}+Q(0)=\alpha IT_{h}+K(T_{h}-T_{c})-0.5I^{2}R+hPL(\frac{T_{c}+2T_{h}}{3}-T_{0}),$$

(25b) $$Q_{c}=Q_{\mathrm{pc}}+Q(L)=\alpha IT_{c}+K(T_{h}-T_{c})+0.5I^{2}R-hPL(\frac{2T_{c}+T_{h}}{3}-T_{0}).$$

The above revised thermoelectric energy equations are derived by taking into account the convective heat transfer between the ambient and the TE materials, which is what distinguishes Equation (25) from the classic thermoelectric energy equations. When the convective term (the fourth term to the right of the equals sign) is removed from the equation, Equation (25) becomes the classic thermoelectric energy equation that is well known in the field and widely used to this day.

It should be noted that the effects of convection have been considered in a number of studies and the subject has been analyzed to varying degrees. Some of the work has been performed with great detail, depth, and theoretical rigor. The work in this paper is simply an exploration of deriving generalized formulas from the fundamental effects of thermoelectrics that are easy to calculate and analyze from an engineering practical perspective (using linear temperature distributions). The work in this paper is rough, but not without practicality and operability, which is of some reference value and significance for the analysis and calculation of specific engineering problems.

**Figure 3:**
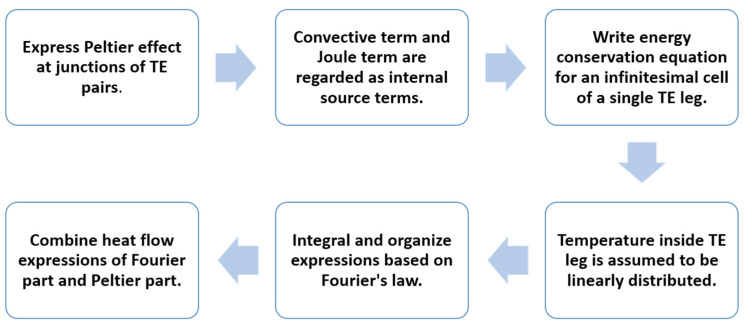
Flowchart of the core steps in deriving the revised equations.

Although the derivation is comprehensive, it may be beneficial to provide a flowchart that summarizes the steps for deriving the revised equations, as shown in Figure 3.

In addition to the various internal energy conversion processes, there are also irreversible heat transfer processes between the TEG and the external heat reservoirs. The expressions for the total heat flow rate (*Q*_H_ and *Q*_C_) of a TEG consisting of multiple TE pairs can be easily written as follows,

(26a) $$Q_{H}=k_{1}S_{1}(T_{1}-T_{h})=nQ_{h}=n[\alpha IT_{h}+K(T_{h}-T_{c})-0.5I^{2}R+hPL(\frac{T_{c}+2T_{h}}{3}-T_{0})],$$

(26b) $$Q_{C}=k_{2}S_{2}(T_{c}-T_{2})=nQ_{c}=n[\alpha IT_{c}+K(T_{h}-T_{c})+0.5I^{2}R-hPL(\frac{2T_{c}+T_{h}}{3}-T_{0})],$$

where *k*_1_ and *k*_2_ are the heat transfer coefficients between the TEG and the high temperature and low temperature heat reservoirs, respectively, *S*_1_ and *S*_2_ refer to the external heat transfer areas of the TEG at the hot and cold ends, respectively, and *n* represents the number of thermoelectric pairs.

## 3. Performance Analysis

### 3.1. Power and Efficiency

The expression for the power output of a TEG consisting of multiple thermoelectric pairs is given by

(27) $$P_{\mathrm{out}}=I^{2}R_{L}={\left(\frac{n\alpha \Delta T}{nR+R_{L}}\right)}^{2}R_{L}$$

where $\Delta T=T_{h}-T_{c}$. As mentioned earlier, the Seebeck effect is the most important thermoelectric effect here, and the product of ΔT and the Seebeck coefficient α equals the magnitude of the voltage produced by this thermoelectric material at this temperature difference.

*P*_out_ can also be expressed in terms of voltage output, i.e.,

(28) $$P_{\mathrm{out}}=V_{\mathrm{out}}I=(n\alpha \Delta T-InR)I=n\alpha I\Delta T-I^{2}nR=\frac{n^{2}\alpha^{2}\Delta T^{2}}{nR+R_{L}}-{\left(\frac{n\alpha \Delta T}{nR+R_{L}}\right)}^{2}nR.$$

Therefore, the energy efficiency of a thermoelectric power generation system is naturally defined as

(29) $$\eta =\frac{P_{\mathrm{out}}}{Q_{H}}=\frac{n\alpha I\Delta T-I^{2}nR}{n[\alpha IT_{h}+K(T_{h}-T_{c})-0.5I^{2}R+hPL(\frac{T_{c}+2T_{h}}{3}-T_{0})]}=\frac{Q_{H}-Q_{C}-nQ_{\mathrm{conv}}}{Q_{H}}=\frac{Q_{h}-Q_{c}-Q_{\mathrm{conv}}}{Q_{h}}.$$

where *Q*_conv_ is employed to represent the convective heat transfer heat flow rate in a single pair of thermocouple legs:

(30) $$Q_{\mathrm{conv}}=2hPL(\frac{T_{c}+T_{h}}{2}-T_{0}).$$

### 3.2. Convective Heat Transfer

The expression for *Q*_conv_ can be given through subtracting Equation (25b) from Equation (25a). The physical meaning of the above expression is so clear that it can be known at a glance without further formula transformations. Equation (30) can be equated to the total convective heat transfer between the surface of the TE leg, where the temperature is equal to (*T*_h_ + *T*_c_)/2 at all locations, and the ambient temperature of *T*_0_.

Similarly, the expression for the convective heat transfer heat flow rate in a single P/N-type TE leg, *Q*_pconv_ or *Q*_nconv_, can be given through subtracting Equation (22b) from Equation (22a) and actually be obtained directly from Equation (11),

(31) $$Q_{\mathrm{pconv}}=Q_{\mathrm{nconv}}=hPL(\frac{T_{c}+T_{h}}{2}-T_{0}).$$

The physical meaning of the above expression is the total amount of convective heat transfer between the surface, with temperature everywhere equal to (*T*_h_ + *T*_c_)/2, of a TE leg (P or N-type), and the ambient with temperature *T*_0_.

The effect of the presence of convective heat transfer on the performance of the TEG can be obtained intuitively from Equation (29). First, the presence of convective heat transfer causes some of the heat that should be transferred to the cold end and partially convertible to electricity to be dissipated directly to the environment. In addition, the presence of the convective term with absolute values greater than zero in the denominator of Equation (29) leads directly to a reduction in the energy efficiency.

In particular, it should be noted that just from the form of Equation (27) or (28) the presence of convective dissipation does not seem to affect the power output, which is clearly inconsistent with the principle of energy conservation. In fact, convective heat transfer certainly affects the temperature distribution (and thus the temperature difference) to varying degrees, which in turn leads to a reduction in power output. It is only in this study that the so-called type I boundary conditions are given directly for TEG with a fixed temperature, considering the practicality of engineering and exploring the derivation of general formulas that are easy to calculate and analyze, so that the effect of convective heat transfer on the temperature (as well as on the power output) is already implied in the given boundary temperature. If the so-called type II boundary conditions with constant heat flux are given for the TEG, the effect of convective dissipation on the temperature distribution and thus on the power output can be seen, but the calculations for this case would be quite complex and not favorable for engineering applications.

### 3.3. Discussion

It is easy to misunderstand the energy transfer process from the form of the thermoelectric energy equation. The factor 0.5 in the Joule term 0.5 *I*^2^*R* does not mean that half of the Joule heat generated inside the thermocouple leg goes to the hot end and the other half to the cold end; it is simply the result of the dual effect of physical homogeneity of the material and integral calculation.

Why someone might “misunderstand” can be better understood by examining the transformation of the convective heat transfer term in the derivation of the revised thermoelectric energy equations. Because of the assumption of a linear temperature distribution on the one hand, and two integrations of the coordinates on the other hand, the convective heat transfer term that finally appears in the revised thermoelectric energy equations does not appear as a factor of 0.5 like the Joule heat term does. In other words, the convective heat transfer terms in Equations (25a) and (25b) do not lead one to “misunderstand” that half of the convective heat transfer (in the form of 0.5*Q*_conv_) is directed to the cold end and the other half to the hot end.

It is very interesting that the expressions for the convective heat transfer terms appearing in Equations (25a) and (25b) are slightly different. The coefficient factor of *T*_c_ in Equation (25a) is one-third while that of *T*_h_ is two-thirds; conversely, the coefficient factor of *T*_c_ in Equation (25b) is two-thirds while that of *T*_h_ is one-third. Because the value of *T*_c_ is less than *T*_h_, the absolute value of the convective heat transfer term in Equation (25a) is actually greater than that of the convective term in Equation (25b). More interestingly, the sum of the absolute values of the convective heat transfer terms in Equation (25a) and Equation (25b) is exactly equal to the amount of convective heat transfer in the entire thermocouple legs expressed by Equation (30).

## 4. Evaluation of the Assumption of Linear Temperature Distribution

**Figure 4:**
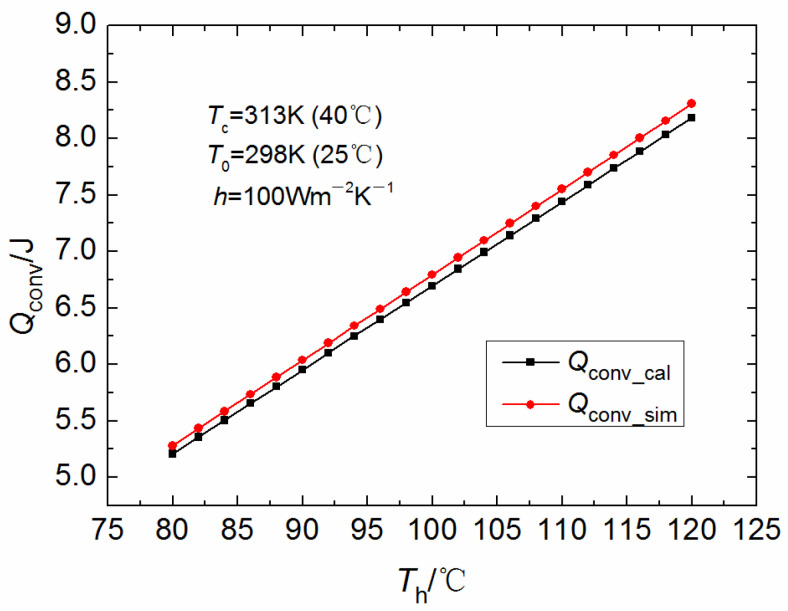
Effect of *T*_h_ on the convective heat dissipation characteristics of the thermoelectric legs.

As mentioned in the derivation of the revised thermoelectric energy equations, the comparison of the results of the formula calculation and the results of the numerical simulation will be made later to verify whether the linear temperature distribution assumption is reasonable. Given that the object of this study is a thermoelectric material with standard geometry, its physical model is clear, and the physical parameters are more available, the numerical simulation is performed by the finite volume method with better conservation. In cases where the research subject is more intricate, the utilization of commercial software based on the finite volume method is also permissible for the execution of numerical simulations. As shown in Figure 4, the convective heat dissipation between the surface of the TE materials and the environment in a typical commercial thermoelectric module (containing 31 pairs of thermoelectric legs) was numerically simulated and the results were compared to those calculated by Equation (30).

The following material properties (bismuth telluride) and geometries obtained from a typical commercial thermoelectric module were used in the numerical simulations and calculations: Seebeck coefficient α = 9.6774 × 10^−4^ VK^−1^, thermal conductance (not thermal conductivity) *K* = 3.84 × 10^−3 ^WK^−1^, electric resistance (one TE pair) *R* = 0.086Ω, number of thermoelectric pairs *n* = 31, and thermoelectric leg dimensions (length × width × height): 2 × 2 × 3 mm.

The calculated convective heat dissipation values increase linearly with increasing hot end temperature, as can be clearly seen from the ramp-up curve in Figure 4 or directly from Equation (30). The simulated convective heat dissipation values also appear to increase almost linearly. Overall, the simulated values are slightly larger than the calculated values at the same temperature, and this difference increases slightly with increasing temperature. Within the computational range of this study, the above difference is controlled to be a little more than 1%. Thus, it is at least shown that the assumption of linear temperature distribution inside the thermoelectric leg is reasonable for low-grade heat utilization (conventional waste heat utilization) at not too high temperatures, and the revised thermoelectric energy equations derived in this paper can be used to meet the accuracy of engineering calculations. From a more detailed analysis, the simulated values at the same temperature are slightly larger than the calculated ones, and the difference increases slightly with increasing temperature, indicating that the real temperature values inside the thermoelectric leg are not strictly linearly distributed, but are slightly higher than the linearly distributed temperature values at all locations.

## 5. Evaluation of the Impact of Convective Heat Transfer

Typically, thermoelectric energy conversion processes are analyzed without considering convective heat dissipation, which is generally considered to be negligible. In order to determine the share of convective heat dissipation in the overall thermoelectric conversion process, the effect of convective heat dissipation on the energy efficiency of the TEG is quantitatively analyzed. The results of the analysis, as shown in Table 1, show that the effect of convective heat dissipation on the efficiency of the TEG increases as the convective heat transfer is gradually enhanced; when the convective heat transfer coefficient is greater than 10 Wm^−2^K^−1^, the effect begins to be non-negligible. From the data, when the convective heat transfer coefficient is less than 10 Wm^−2^K^−1^, i.e., when the TEG is under natural convection (mostly indoors), the effect of convective heat dissipation can be neglected; however, when the TEG is under forced convection (mostly outdoors), and especially when the convective heat transfer coefficient is much higher than 10 Wm^−2^K^−1^, convective heat dissipation is a factor that must be considered to affect the system performance. It should be noted that this study was conducted on a commercial thermoelectric module of relatively small size; for larger TEG units (especially customized ones), where the thermoelectric material used has a larger volume and surface area, careful insulation is required to minimize the effect of convective heat dissipation on performance. Table 1 also shows that the relative effect of convective heat dissipation on the system efficiency decreases with increasing temperature, which reminds us that thermal insulation should not be neglected when using TEG for low-temperature waste heat utilization.

## 6. Conclusions

A thorough examination of the classic thermoelectric effects and heat transfer theory has yielded a revised set of thermoelectric energy equations. These equations are notable for their incorporation of convective heat dissipation from the surface of thermoelectric materials to the ambient. The revised thermoelectric energy equations have a very simple form, which is very convenient for engineering calculation and analysis.

Furthermore, it has been demonstrated that the relative impact of convective heat dissipation on system efficiency diminishes with increasing temperature. On average, for every 20-degree decrease in hot-side temperature, there is a 0.8-percent-point decrease in system efficiency for an equivalent level of convective dissipation. This observation underscores the necessity of prioritizing thermal insulation in the utilization of TEG for low-temperature waste heat management.

A comparison of the results of formula calculation and numerical simulation was conducted to verify the reasonableness of the linear temperature distribution assumption in the derivation process. Within the computational range of this study, the differences between the simulated values and the calculated values are controlled to be a little more than 1%, which is sufficient to meet the needs of engineering calculation and analysis.

The question of whether the linear temperature distribution assumption is applicable in the application of various types of thermoelectric materials, or how much error the application of the linear temperature distribution assumption introduces into the analysis of various types of thermoelectric materials, as well as the assessment of the adaptability of the above findings to a wider range of application temperatures, will be an important aspect of the subsequent research.

## Figures and Tables

**Table 1:** Effect of *h* on the energy efficiency of the thermoelectric generator.

*h* (Wm^−2^K^−1^)	*T*_h_ = 80 °C		*T*_h_ = 100 °C		*T*_h_ = 120 °C	
	η	Δη% *	η	Δη%	η	Δη%
0	1.91	0	2.82	0	3.71	0
10	1.84	−3.7	2.74	−2.8	3.61	−2.7
100	1.4	−26.7	2.15	−23.8	2.9	−21.8

* compared to η at *h* = 0.

## Data Availability

The original contributions presented in this study are included in the article. Further inquiries can be directed to the corresponding author.

## Nomenclature

*h*	heat transfer coefficient (Wm^−2^K^−1^)
*I*	current (A)
*k*	thermal conductivity (Wm^−1^K^−1^), heat transfer coefficient (Wm^−2^K^−1^)
*K*	thermal conductance (WK^−1^)
*L*	length (m)
n	number
*P*	circumference (m), power (W)
*Q*	heat transfer or flow rate (W)
*R*	resistance (Ω)
*S*	area (m^2^)
*T*	temperature (K)
*V*	voltage (V)
*x*	coordinate (m)
Greek letters	
α	Seebeck coefficient (VK^−1^)
ρ	resistivity (Ωm)
η	efficiency
Subscripts	
0	ambient
1	high temperature heat reservoir side
2	low temperature heat reservoir side
c/C	cold
conv	convection
h/H	hot
L	load
n/N	N-type thermoelectric material
out	output
p/P	P-type thermoelectric material
pc	Peltier at cold-end
ph	Peltier at hot-end
TE	thermoelectric
TEG	thermoelectric generator
